# How to Effectively Encourage Sustainable Food Choices: A Mini-Review of Available Evidence

**DOI:** 10.3389/fpsyg.2020.589674

**Published:** 2020-11-16

**Authors:** Wokje Abrahamse

**Affiliations:** School of Geography, Environment and Earth Sciences, Victoria University of Wellington, Wellington, New Zealand

**Keywords:** sustainable food choices, interventions, nudges, prompts, information provision, social norms

## Abstract

Food choices are difficult to change. People’s individual motivations (such as taste, cost, and food preferences) can be at odds with the negative environmental outcomes of their food choices (such as deforestation, water pollution, and climate change). How then can people be encouraged to adopt more sustainable food choices? This rapid review uses a dual-processing framework of decision-making to structure an investigation of the effectiveness of interventions to encourage sustainable food choices (e.g., local and organic food consumption, reducing meat and dairy intake, reducing food waste) via voluntary behavior change. The review includes interventions that rely on fast, automatic decision-making processes (e.g., nudging) and interventions that rely on more deliberate decision-making (e.g., information provision). These interventions have varying degrees of success in terms of encouraging sustainable food choices. This mini-review outlines some of the ways in which our understanding of sustainable food choices could be enhanced. This includes a call for the inclusion of possible moderators and mediators (past behavior, attitudes, beliefs, values) as part of effect measurements, because these elucidate the mechanisms by which behavior change occurs. In light of the climate change challenge, studies that include long-term effect measurements are essential as these can provide insight on how to foster sustained and durable changes.

## Introduction

Encouraging people to adopt environmentally sustainable diets is an important step toward lowering greenhouse gas emissions. Several studies indicate that individual food-related behaviors–adopting plant-based diets, buying foods with a low carbon footprint, recycling of edible food waste–have significant impacts on overall emission reductions (Steinfeld et al., 2006; Parfitt et al., 2010; Berners-Lee, 2011; Garnett, 2013, 2016; Gerber et al., 2013; Hoolohan et al., 2013; Godfray et al., 2018). This suggests that encouraging the uptake of environmentally sustainable food behaviors can have a substantive impact on limiting climate change.

Encouraging people to alter their food choices is notoriously difficult (Nestle et al., 1998). Large-scale initiatives, such as the “5-a-day” campaign promoting fruit and vegetable intake, are well-known among the general public, but have not necessarily resulted in substantive changes in behavior (Wood, 2019). In the field of environmentally sustainable food choices, a growing body of intervention studies can help shed light on the efficacy of behavior change interventions.

This integrative mini-review (note that this is not a systematic literature review) summarizes what is known about the effectiveness of interventions to encourage environmentally sustainable food choices. It draws on a range of peer-reviewed studies, from randomized control trials to pre-test/post-test design, conducted in a variety of settings, from university cafeterias to convenience stores, and a variety of food-related behaviors. The methods that were used to select the studies, including keyword searches, inclusion and exclusion criteria, and the time period covered, can be found in the Supplementary Materials. The two overarching questions of this review are: how effective are behavior change interventions to encourage environmentally sustainable food choices and what psychological mechanisms can account for the effects?

## Theoretical Framework

In psychology, human behavior is often explained via dual-process theories of behavior (Evans, 2008). Dual-process theories of behavior posit that there are two distinct processes that govern decision-making. One is automatic, quick and unconscious, the other is deliberative, slow, and conscious (Kahneman, 2011). Some decisions are informed by the central route (requiring cognitive effort) and other decisions are guided by the peripheral route of information processing (based on cues and heuristics) (Petty and Cacioppo, 1986).

Several scholars argue that food choices are habitual: they are automatic responses to cues in the environment (Wood and Neal, 2009; Lally et al., 2010; Neal et al., 2011). Neal et al. (2011) found that when people in a cinema were given a box of 7 days old stale popcorn, those with strong popcorn eating habits ate 70% of the stale popcorn. What is more, nobody liked the stale popcorn. The cue (watching a movie in a cinema) made the response (eating popcorn) more or less automatic, regardless of people’s motivations (disliking the popcorn). Other researchers propose that food choices are (also) driven by a deliberate decision-making process. For example, a recent meta-analysis found that people’s organic food choices are strongly associated with attitudes toward organic foods as well as past behavior (a proxy for habits) (Nardi et al., 2019).

The distinction between fast and slow decision-making processes could help explain the (in)effectiveness of behavior change interventions. For example, one of the reasons why the “five-a-day” campaign may not have resulted in increased fruit and vegetable consumption is that this type of information provision relies on the slow mode of processing. If fruit and vegetable consumption is indeed habitual behavior, information provision will not change people’s behavior. Rather, cues in the environment could be altered (placing fruits by the check-out counter; see Kroese et al., 2016) to facilitate the desired behavior.

This integrative mini-review (please see Supplementary Materials for methods detailing study selection) uses a dual-processing framework to structure an investigation of the effectiveness of interventions to encourage environmentally sustainable food choices. The review includes nudging, food labels, visual prompts, information provision, and the use of social norms (for detailed information about each study, see Table 1). This review has two main aims: (i) examine the effectiveness of behavior change interventions and (ii) explore underlying psychological mechanisms that can help explain why an intervention is (in)effective. In doing so, this review summarizes recent advances and the current state of our understanding in the field.

**TABLE 1:** Summary table of key characteristics of the intervention studies included in this mini-review.

References	Country	Intervention	Study design	Sample characteristics	Duration of intervention	Outcome measure(s)	Effect	Mediator/Moderator
Bernstad et al. (2013)	Sweden	Information provision	Between-subjects design: 1. Written information 2. Written + face-to-face communication	Residents *N* = 680	104 weeks	Amount (kg) of correctly recycled food waste	Face-to-face communication was associated with increase in food waste recycling after 8 months, effect diminished after 18 months	Not included
Bernstad (2014)	Sweden	Information provision (written)	Pre-post design: 1. Written information 2. Food waste equipment in kitchens	Households *N* = 1632	10 weeks	Amount (kg) of correctly recycled food waste	Information was not associated with an increase in food waste recycling, but recycling equipment was	Not included
Brunner et al. (2018)	Sweden	Carbon labels (traffic light system: red = high; yellow = medium; green = low impact)	Pre-post design	Students; *N* = 3,715	Baseline: 28 days; Intervention: 33 days	Type of dish chosen as function of type of carbon label	An 11.5% increase in sales of green-labeled dishes. No difference in yellow- or red-labeled meat dishes. Labels associated with 3.6% emission reduction	Gender and age (moderators): No gender and age differences in response to carbon label
Carfora et al. (2019)	Italy	Text messages about health and/or environmental benefits Self-monitoring Goal setting	RCT 1. Control 2. Health benefits 3. Environment benefits 4. Health + environment	Students; *N* = 261	2 weeks + 1 month follow-up	Red meat consumption; attitudes toward reducing red meat consumption	Health and environment messages associated with lower red meat consumption after 1 month. No added effect of combined message	Effect of the intervention on meat consumption was mediated by attitudes toward reducing red meat
dos Santos et al. (2018)	Denmark	Nudging	Quasi-experimental 1. Control 2. Nudge (dish of the day)	Adolescents *N* = 94 Elderly *N* = 97	4 months	Plant-based meal choice in cafeteria	No difference between control and nudge group in plant-based meal choices	Possible moderators were included, but not tested
Elofsson et al. (2016)	Sweden	Carbon label	RCT 1. Standard label 2. Climate certified label	Shoppers at 17 supermarkets	4 weeks	Sales of climate-certified milk	An 6–8% increase in sales of milk when it carried a “climate-friendly” label, relative to a standard label	Not included
Garnett et al. (2019)	United Kingdom	Nudging	RCT 1. Control 2. Nudge (increased availability)	*N* = 94,644 meals (3 cafeterias)	44 lunchtimes during the autumn term	Vegetarian meal choice	Doubling of vegetarian availability (from 25 to 50%) increased portion of vegetarian sales from 19.1–26.9%	Past behavior was a significant moderator. The impact of increased availability was stronger among those who were not normally eating vegetarian options
Kurz (2018)	Sweden	Nudging	Quasi-experimental 1. Control 2. Nudge (menu order and dish visibility)	Staff and students *N* unknown (sales data was used)	10 months (1 academic year)	Vegetarian meal choices	Nudge associated with higher vegetarian meal choice (from 14 to 20%)	Not included
Linder et al. (2018)	Sweden	Information provision	RCT 1. Control 2. Information leaflet	Households; *N* = 264	1 leaflet, 2 years of data collection	Food waste (in kilograms)	Households in intervention group significantly reduced food waste relative to control up to 8 months after leaflet distribution	Not included
Monroe et al. (2015)	United States	Information provision + goal setting (web-based)	Quasi-experimental 1. Control 2. Information	Students *N* = 607	5 weeks	Self-reported green eating behaviors	Intervention group: small but significant increase in green eating behaviors Control group: no change	Not included
Nomura et al. (2011)	United Kingdom	Social comparison feedback	RCT 1. No-treatment control 2 Social comparison feedback	Households; *N* = 9,082	2 months	Participation in food waste recycling scheme	Participation rates in treatment group increased by 0.5% after 1 month and by 2.8% after 2 months relative to control group	Not included
Shearer et al. (2017)	United Kingdom	Visual prompt	RCT 1. Control 2. Sticker on waste bin	Households; *N* = 64,000	Baseline (13 weeks); sticker (15 weeks)	Weight of collected food waste	Visual prompt increased food waste recycling by 20% relative to control	Not included
Spaargaren et al. (2013)	The Netherlands	Climate labels	Pre-post: 1. Baseline (no label); 2. “light” climate label; 3. “comprehensive” climate label + information	Patrons of a university canteen	Baseline: 5 weeks; “light” label: 10 weeks comprehensive label: 8 weeks	Sales data of lunch meals	A small but statistically significant 3% shift toward lower carbon lunches	Not included
Sparkman and Walton (2017; study 4)	United States	Dynamic social norms + $5 discount on lunch	Quasi-experiment 1. Control 2. Descriptive norm 3. Dynamic norm	Patrons of a university café *N* = 304	2 days	Sales of meatless lunches	Sales significantly higher for dynamic norm (34%), compared with descriptive norm (17%) and control (21%). No difference between control and descriptive norm	Not included
Stöckli et al. (2018)	Switzerland	Prompts	Between-subjects design 1. Control 2. Prompt 3. Normative prompt	Patrons of a pizzeria *N* = 54	6 weeks; observation period of 90 min each day	Whether people disposed of or took away pizza leftovers	Both prompts had a small effect on waste behaviors; but no differences between prompts	Not included
Sussman and Gifford (2013)	Canada	Prompts, modeling	Quasi-experimental design 1. Control 2. Prompt 3. Models 4. Sign + models	Diners at shopping mall food courts *N* = 562	2 days	Correct composting behavior	Modeling was associated with a significant (14%) increase in composting behavior. Sign was not associated with a change in composting	Not included
Sussman et al. (2013)	Canada	Prompts, modeling	Pre-post design 1. Baseline 2. Prompt 3. One model 4. Two models 5. Prompt	Patrons at university cafeteria *N* = 1,066	4 weeks	Correct composting behavior	Composting increased from 12.5% (baseline) to 21% (prompt), 25% (one model) and 42% (two models)	Not included
Vanclay et al. (2011)	Australia	Carbon labels	Pre-post design	Convenience store customers (*N* unknown); sales of 2,890 items	Baseline: 4 weeks; intervention: 8 weeks	Point-of-sale data for milk, spreadable butter, canned tomatoes, bottled water, pet food	A 5% increase in sales of low-carbon foods. Significant 20% increase in sales when low-carbon items were also cheapest	Not included
Vandenbroele et al. (2018)	Belgium	Nudging: reduced portion size	Field experiment 1. Control (larger portion) 2. Nudge (smaller portion)	Customers in retail store	1 month	Sales data of meat sausages	Higher sales (52%) of smaller portion relative to control (48%)	Not included
Visschers and Siegrist (2015)	Switzerland	“Climate-friendly” label	Pre-post 1. Baseline 2. Climate-friendly label + information posters	Staff and students at a university cafeteria	Baseline: 5 days Intervention: 17 days	Sales of climate-friendly meals	Sales of “climate-friendly” meals increased by 10%	Not included
Vlaeminck et al. (2014)	Belgium	Environmental label based on composite score (carbon, land use, water)	Between-subjects: 1. Default label 2. Difficult to understand label 3. Easy to understand label	Supermarket; *N* = 150	9 days (the three labels were switched at random)	Sales of protein (stead, chicken, veggie burger), tomatoes and apples	Environmental labels increase eco-friendliness of food choices by 5.3% relative to default. No impact of eco-labels on sales in protein category	Not included
Whitehair et al. (2013)	United States	Visual prompt	Pretest-posttest 1. Baseline 2. Prompt 3. Feedback	Students *N* = 540	6 weeks (2 weeks baseline; 2 weeks prompt, 2 weeks feedback)	Edible food waste	Prompts significantly reduced edible food waste by 15%; no effect of feedback	Environmental beliefs—but no effect
Zhou et al. (2019)	United Kingdom, France, Denmark, Italy	Nudging	RCT 1. Control 2. Nudge (“dish of the day”)	People aged 65 or over *N* ranged between 47 and 118	6 months	Plant-based meal choice	Making plant-based option dish of the day (nudging) was not associated with meal choices in any of the countries	Universalism values were positively associated with choosing plant-based meals, irrespective of the intervention

## Overview of Behavior Change Interventions

### Nudging

Nudges involve a (simple) change to the context in which people make decisions (Thaler and Sunstein, 2009). Nudges do not change economic incentives or ban certain products. Rather, nudges steer people toward the desired behavior by changing the choice architecture. Different types of nudges have been implemented in food research, including changes to the default (e.g., labeling a vegetarian option the “dish of the day”) and changes to the food environment (e.g., placing healthy foods by the check-out counter instead of unhealthy foods; increasing the availability of vegetarian options on a menu).

The assumptions that underlie nudging are grounded in behavioral economics. Behavioral economics identifies common patterns of thinking that deviate from the assumption that people are rational decision makers (Sunstein, 2014). Nudging interventions alter the choice architecture (e.g., the food environment) so that people’s automatic, quick mode of decision making is activated. This suggests that nudging might be particularly effective in changing behaviors that rely on automatic processes, such as food choices (van Kleef and van Trijp, 2018; Vecchio and Cavallo, 2019).

#### Changes to the Default

One version of nudging consists of labeling a specific menu item as “dish of the day,” or “Chef’s recommendation.” While scenario studies (involving hypothetical meal choices) have shown promising effects of this type of nudging on vegetarian meal choices (e.g., Campbell-Arvai et al., 2014; Bacon and Krpan, 2018), experimental field studies do not seem to observe significant effects. In a randomized controlled field experiment conducted in four European countries, Zhou et al. (2019) found that labeling plant-based options as “dish of the day” did not influence people’s meal choices in a restaurant setting. Study findings by dos Santos et al. (2018) also indicate that a “dish of the day” nudge in cafeterias did not increase the uptake of plant-based meals.

#### Changes to the Food Environment

Other applications of nudging involve changing something in the food environment to encourage sustainable food choices. Kurz (2018), for example, found that when the vegetarian option on a menu was made more visible (putting it on the counter where customers placed their order) sales of vegetarian dishes showed a small but significant increase relative to baseline. Altering the availability or portion size is another form of changing the food environment. Garnett et al. (2019) varied the vegetarian offerings in three University of Cambridge college cafeterias and collected individual-level meal selection data. A doubling of the availability of vegetarian offerings (from 25 to 50%) was associated with an 8% increase in sales, compared with a control group. Similarly, Vandenbroele et al. (2018) found that adding smaller portion sizes to a retailer’s assortment reduced the total volume of meat sold, relative to a control retailer.

### Carbon and Environmental Labels

Studies show that people are generally unaware of the extent to which their food choices impact the environment (e.g., de Boer et al., 2016). Carbon labels can provide insight into the climate impact of a particular food. Environmental (or eco) labels provide a holistic overview of impacts, such as land use changes, deforestation, water use, pesticide use and greenhouse gas emissions. These environmental impacts are often estimated via Life Cycle Analysis (LCA), whereby impacts associated with all phases of a product’s life cycle (production, distribution, consumption, and disposal) are added up (see for example Berners-Lee, 2011; Berners-Lee et al., 2012; Hallström et al., 2015). Food labels are a type of information provision that guide food choices in the food environment, when people make decisions about which product to buy.

Researchers have proposed that food labels affect people’s food choices by virtue of being an environmental label (e.g., Vlaeminck et al., 2014). Such labels might “prime” people to choose an environmentally friendly food product via a quick, unconscious decision-making process. Guéguen et al. (2012), for example, found that when menus contained watermark visual cues related to the sea, diners were more likely to choose fish dishes. Other scholars, in contrast, would suggest that a more conscious and deliberate process is involved: carbon labels activate people’s environmental values and beliefs, which in turn influence food choices. Empirical studies indicate that the effect of carbon labels on food choices depends on people’s levels of environmental concern (e.g., Thøgersen, 2000; Grunert et al., 2014; Shewmake et al., 2015; Thøgersen and Nielsen, 2016).

Food labels seem to have a positive, but modest effect on people’s food choices (see Table 1). In a randomized field experiment in Swedish retail stores, Elofsson et al. (2016) found that when milk carried a “climate certified” label, daily sales increased by approximately 6% relative to a standard milk label. A study in an Australian convenience store by Vanclay et al. (2011) observed an increase in sales of food products that carried a “green” low carbon label, relative to products with a higher carbon impact. However, this study also found that carbon labels did not necessarily discourage consumers from buying products with a high climate impact, such as milk (see also Vlaeminck et al., 2014 for a similar finding).

Carbon labels have also been used alongside other interventions (Spaargaren et al., 2013; Visschers and Siegrist, 2015; Brunner et al., 2018). For example, Brunner et al. (2018) developed carbon labels (using a green/yellow/red traffic light system to indicate climate impact) for dishes in a university student cafeteria in Gothenburg, Sweden. In addition, information about links between food and climate change was provided via a website and posters in the cafeteria. While there was a significant 11.5% increase in sales of climate-friendly green dishes during the label phase (compared with baseline), there were no changes in sales of yellow-or red-labeled meat dishes (i.e., dishes with a higher climate impact). Because a combination of information provision and food labels was used in these studies, it is difficult to attribute any effect of the use of food labels alone.

### The Provision of Information

In contrast to food labels, which guide food choices “in the moment” (i.e., when people are in a supermarket), information provision generally occurs outside the food environment. This can be, for example, mass media information campaigns, or guidelines from the government (e.g., the ever-changing “food pyramid”; see Nestle, 2013). The provision of information or education is based on a “knowledge-deficit” approach and assumes that when people have more information and “better” knowledge, that behavior change will follow. As such, information provision generally assumes a deliberate, conscious decision-making process.

For example, Monroe et al. (2015) developed an interactive web-based intervention to encourage the uptake of environmentally friendly eating behaviors among university students. The intervention consisted of modules on local food, food waste, and environmentally friendly protein and was displayed as text, pictures, video clips and interactive quizzes. A significant increase in self-reported green eating behaviors was observed, relative to a control group. Carfora et al. (2019) found that text messages about health or environmental benefits (combined with a reminder to reduce meat consumption) were associated with a reduction in self-reported red meat consumption immediately following the intervention and a follow-up 1 month later.

Bernstad et al. (2013) found that while written information was not effective in encouraging food waste recycling, when the same information was delivered in a face-to-face format, it did change behavior. In a separate study, Bernstad (2014) found that written information did not result in behavior change, but the subsequent installation of waste sorting equipment was associated with a significant 49% increase in the amount of recycled food waste. Linder et al. (2018) developed information that specifically addressed key barriers to recycling food waste (based on interviews with residents) and found that the provision of targeted information was associated with a significant 26% increase in food waste recycling (relative to baseline).

### Visual Prompts

Visual prompts are a brief form of information provision that act as a reminder to engage in a certain behavior (e.g., stickers, posters, signs, flyers). Prompts appear to be most effective when the behavior is easy to do (Abrahamse and Matthies, 2018) and when people are already motivated to perform the behavior (Schultz, 2014). Prompts can act as cues and promote behaviors via a quick decision-making process. Indeed, some researchers refer to prompts as “nudges” (e.g., Shearer et al., 2017).

In a randomized control trial, Shearer et al. (2017) found that placing a sticker on general waste bins reminding people to recycle their food waste (“No food waste please. Remember to use your food recycling caddy”) increased the amount of recycled food waste by 20%, relative to a control group (no sticker on bin). Whitehair et al. (2013) examined the effectiveness of a visual prompt to reduce edible food waste in a university dining facility. When a visual prompt was introduced (reminding students to not waste food), the amount of edible food waste was reduced by 15%. When information was then provided on how much food waste was generated in the cafeteria, this did not have an additional effect. This may be because telling students that a lot of food is wasted may have (inadvertently) made a social norm salient (cf. Cialdini, 2003) that everybody wastes food. Sussman et al. (2013) observed that a visual prompt was associated with a significant increase in composting behavior in a repeated measures study. In a between-subjects study, however, they found that a prompt did not influence composting behavior (Sussman and Gifford, 2013).

### Social Norms

Social norms refer to the notion that behavior is influenced by what other people do (descriptive social norms) and what people think is expected of them (injunctive norms) (Cialdini, 2003). Social norms influence behavioral choices when they are made salient. There is some evidence to suggest that people differ in the degree to which they are susceptible to social norms (e.g., Stöckli and Hofer, 2020). This implies that people may not necessarily follow social norms because these norms are “cued,” but because they are important to people in their deliberate decision making.

Social norms are used as part of information provision or feedback provision and sometimes as part of short prompts. Sparkman and Walton (2017) used social norms as part of information provision to encourage a reduction in meat consumption in a campus cafeteria. The authors examined the effect of descriptive social norms (the % of other people who do a behavior) with so-called “dynamic” social norms (norms about the changes in behavior other people engage in). Patrons who were given information about dynamic norms (“30% of Americans have started to make an effort to limit their meat consumption”) were significantly more likely to choose a meatless lunch, compared with a descriptive social norm message and control. Stöckli et al. (2018) found that a standard prompt (encouraging people to ask for a takeaway box for any leftovers) and a prompt with a descriptive norm message (i.e., “many guests ask us to wrap their pizza leftovers”) were associated with an increase in patrons asking for takeaway boxes. The normative prompt was no more effective than the standard prompt.

Nomura et al. (2011) conducted a randomized control trial to examine the effect of social norm feedback on participation in a food waste reduction scheme. They found that households in the social norm group significantly increased participation rates, relative to a control group. Households who had received feedback accompanied by a smiley face (the street performed better than average) and those who had received a sad face (the street performed worse than average) had higher participation rates relative to control streets.

## Psychological Mechanisms: Exploring Mediators and Moderators

It is important to consider the psychological mechanisms through which interventions result in behavior change. Relatively few studies in this review included potential moderators or mediators and what follows illustrates what some of these mechanisms might be.

### Past Behavior

Past behavior refers to the extent to which people engage in the target behavior prior to the intervention. In some cases, frequency of past behavior is used as an indicator of habit. Garnett et al. (2019) found that past behavior was a significant moderator of the effect of nudging on food choices. The effect of the nudge (increased availability of vegetarian meal choices) was stronger for those who would not normally eat vegetarian options. Scenario studies point to a similar effect. For example, Bacon and Krpan (2018) found that labeling a vegetarian option as “Chef’s Recommendation” (nudge) did not affect vegetarian meal choices, but the effect of nudging was moderated by past behavior. Infrequent vegetarians were more likely to choose the vegetarian option when this was presented as the recommended option, compared with frequent vegetarians.

### Universalism Values

Studies have found that human values (i.e., guiding principles in people’s lives; Schwartz, 1994) are associated with sustainable food choices. Universalism values, for example, are (positively) associated with organic food choices (Vermeir and Verbeke, 2008) and vegetarianism (Hayley et al., 2015; Graham and Abrahamse, 2017). Universalism values are part of the self-transcendence dimension and reflect the value people place on care for nature.

Some studies have found that universalism values are predictive of food choices independently of the effect of an intervention (e.g., Campbell-Arvai et al., 2014; Zhou et al., 2019) Zhou et al. (2019) found that participants with stronger universalism values were more likely to choose plant-based options, irrespective of a nudge intervention. This suggests, perhaps, that for people with strong universalism values, the choice of plant-based meals is the result of deliberate decision-making and not easily changed by an intervention that relies more on automatic decision making.

It might be that values moderate the effect of an intervention on sustainable food choices, but the evidence for this is limited. Interventions to encourage sustainable food choices may well be more effective when people have stronger universalism values. For example, Graham and Abrahamse (2017) found that an informational message about the climate impacts of meat consumption was associated with stronger intentions to reduce meat consumption, particularly for people with strong self-transcendence values. However, none of the intervention studies included in this review reported possible moderating effects of universalism (or other) values.

### Attitudes and Beliefs

The literature points to a close connection between people’s attitudes and beliefs and their food choices. Carfora et al. (2019) found that attitudes toward red meat mediated the effect of their text message intervention on red meat consumption. These text messages were associated with a more positive attitude toward reducing the consumption of red meat. This strengthened attitude, in turn, was associated with a reduction in red meat consumption. Lab studies have also found evidence for a mediating role of attitudes and beliefs. For example, Vainio et al. (2018) found that people’s prior beliefs about meat influenced the effectiveness of an informational message. Reading an informational message was only associated with stronger behavioral intentions among those who already held strong negative beliefs about meat (“meat-skeptics”) and not among so-called “meat believers.”

## Discussion

The findings of this review indicate that interventions can be used effectively to encourage environmentally sustainable food choices. The review draws on a substantive body of research on this topic. This ranges from carefully crafted interventions that focus on people’s motivations and deliberate decision-making processes to interventions that involve simple changes in the choice architecture that facilitate certain behaviors in more “cued” and unconscious ways.

Nudging interventions have some potential to encourage sustainable food choices. Increasing the availability of vegetarian dishes was shown to be effective (Garnett et al., 2019) and so was a reduction in portion sizes of meat (Vandenbroele et al., 2018). However, a “dish of the day” approach seemed ineffective (Zhou et al., 2019). Overall, food labels are effective in encouraging sustainable food choices on their own (e.g., Vanclay et al., 2011) and as part of wider information campaigns about links between food and climate change (e.g., Spaargaren et al., 2013; Visschers and Siegrist, 2015). But it would appear that carbon labels do not necessarily discourage the uptake of products with a high climate impact, such as milk or meat (e.g., Vanclay et al., 2011; Vlaeminck et al., 2014; Brunner et al., 2018).

The provision of information alone is generally not considered to be an effective strategy for behavior change more generally (Abrahamse et al., 2005; Schultz, 2014). The findings in the area of sustainable food corroborate this (e.g., Bernstad, 2014). However, when information is crafted to address specific behavioral barriers (Linder et al., 2018), when it is combined with a motivational goal (Monroe et al., 2015; Carfora et al., 2019) or when it emphasizes social norms (Sparkman and Walton, 2017), information provision can have a positive impact.

The evidence-base for the effectiveness of interventions to encourage environmentally sustainable food choices is growing. However, more research is needed on possible mediators and moderators that can explain why a behavior change intervention was successful (or not). Including moderators or mediators, such as past behavior, cultural values, and prior beliefs and attitudes can provide valuable insights into the mechanisms by which interventions change behavior.

Only one intervention study (Zhou et al., 2019) examined cross-country differences in the effectiveness of a behavior change intervention (a “dish-of-the-day” nudge). While the nudge intervention was not effective in any of the four countries, the authors did observe that participants from the United Kingdom more often tried plant-based dishes compared with French participants. This could for example be due to a higher prevalence of vegetarianism in the United Kingdom relative to France. More research is needed to explore the role of social and cultural processes and how they are linked to food choices (for a review on this topic, see Carrus et al., 2018). More comparative research would also be useful to better understand the effectiveness of different interventions in different food environments (e.g., at home vs. a restaurant vs. a supermarket).

Lastly, relatively little is known about the long-term effects of interventions, as a majority of studies measured immediate, short term effects only. More research could be conducted on the durability of behavior change. Increasing the availability of vegetarian options may be effective in the short term (e.g., at the point of sale), but it is not clear whether this “nudge” will have the potential to affect behavior in other settings, or to instill durable changes (see also Ewert, 2020). This is an important area for future research, because moving toward the adoption of lower carbon diets will require sustained changes in behavior.

## Author Contributions

WA conducted the literature search, conducted the literature review, and wrote the manuscript.

## Conflict of Interest

The author declares that the research was conducted in the absence of any commercial or financial relationships that could be construed as a potential conflict of interest.
