# Supplementary material for: How to Effectively Encourage Sustainable Food Choices: A Mini-Review of Available Evidence
Source: Front Psychol. 2020 Nov 16;11:589674. doi: 10.3389/fpsyg.2020.589674 (PMC7701282; doi:10.3389/fpsyg.2020.589674)
Supplement: Supplementary file 1 [file Data_Sheet_1.docx]

**Supplementary material: Methods**

*Selection procedure*

Studies were located by searching for relevant keywords in Web of Science, Scopus, Google Scholar as well as journals in the field of social environmental psychology and food studies (e.g., Journal of Environmental Psychology, Appetite). In addition, the reference lists of retrieved articles were used to locate any additional publications. The keywords included combinations of interventions (e.g., nudge/dish of the day/choice architecture, prompts, information) and behaviours (e.g., organic food, vegetarianism, plant-based meals). After scanning abstracts and methods sections for relevance and the evaluation criteria (see below for details), a total of 23 intervention studies spanning the time period between 2011 and 2020 were obtained. The cut-off date for the search was July 1^st^, 2020. Please note that this paper is a rapid review as part of a Frontiers in Psychology mini-review format. As such, it does not represent a systematic literature review.

This mini-review focuses on environmentally sustainable food choices. In this paper, environmentally sustainable food choices are understood to mean food choices that have a low(er) environmental impact. This could happen through reduced greenhouse gas emissions (plant-based diets versus meat-based diets; throwing away less food that ends up in landfill, thereby reducing methane emissions), lower levels of (water) pollution, and lower use of harmful substances such as pesticides (organic foods versus conventionally grown foods). For this review, studies focused on the following behaviours: organic food choices, choosing climate-certified food products, choosing products with an eco-label, recycling of food waste, eating plant-based (vegan) or vegetarian meals, and different ways of reducing meat consumption (eating less meat per meal, or eating meat less frequently per week).

The interventions are classified and described according dual process models of behaviour change (Evans, 2008). Based on this, certain intervention can be considered to encourage fast, automatic decision-making (such as nudging, or visual prompts) whereas other interventions are based on slow, deliberate decision-making (e.g., information provision). Behaviour change interventions included in this review are aimed at encouraging voluntary behaviour change, by targeting an individual’s perceptions, preferences and abilities. For this reason, economic (such as pricing policies) and regulatory interventions were excluded from this mini-review

In this mini-review, one of the challenges was how to classify the interventions.

For example, the work by behavioural economists Sunstein and Thaler classifies the use of social norm interventions as ‘nudging’, whereas the psychology literature tends to refer to them as social norm interventions. Similarly, information provision is referred to as a nudge in Sunstein (2014), whereas other scholars would consider the provision of information perhaps as an example of a more conscious, deliberate path of behaviour change (via the knowledge-deficit approach; see Schultz, 2014). The terms that are used to describe and classify the interventions are those used by the study authors. If the authors have named their intervention a ‘visual prompt’, then that is the term used in this review (rather than a nudge).

This review focuses on interventions that are dedicated to changing behaviour for reasons of environmental sustainability. Choosing environmentally sustainable foods often comes with a trade-off between individual benefits and collective environmental outcomes; this is different from for example changing food choices for health concerns. This suggests that the motivational process for changing environmentally sustainable food choices may be different relative to changing health-related food choices. Therefore, intervention studies focused on health aspects associated with food (for example reducing meat consumption because of the link to cardiovascular disease) were excluded from this mini-review. Health interventions have been reviewed elsewhere (e.g., Bucher et al. 2016; Taufik et al. 2019; Vecchio and Carvallo, 2019).

*Evaluation criteria*

To assess the effectiveness of interventions aimed at encouraging environmentally sustainable food choices, the following criteria were considered. Intervention studies were included when:

1. The outcome measure consisted of a behavioural measure (observed or self-reported). This meant that studies focusing on attitudes and/or behavioural intentions were excluded.

2. The study involved a research design whereby the effect of the intervention was measured against another (control) group, and/or a baseline measure; this includes randomised control trials, quasi-experimental designs, and repeated measures designs (pre-test/post-test). This means that correlational studies were excluded from this review.

3. The intervention was applied to a real-world setting (e.g., restaurant, household). This means that studies that measured sustainable food choices as part of hypothetical choice scenarios (e.g., lab studies) were excluded.

Table 1 (see Supplementary Materials) lists the intervention studies included in this mini-review. The table details the study authors, country, intervention type, sample and sample size, duration of the intervention, effects, and inclusion of mediator and moderator variables.
